# Gourd-shaped transmylohyoid dermoid cyst occurring in the oral floor: a case report

**DOI:** 10.1093/jscr/rjag670

**Published:** 2026-07-31

**Authors:** Yunosuke Ikeda, Atsushi Ogawa, Tadashi Kawai, Isao Hoshi, Taifu Hirano, Mitsuru Izumisawa, Hiroyuki Yamada

**Affiliations:** Division of Oral and Maxillofacial Surgery, Department of Oral and Maxillofacial Reconstructive Surgery, School of Dentistry, Iwate Medical University Dental and Oral Surgery, 020-8505 Morioka, Japan; Dentistry and Oral Surgery, Iwate Prefectural Kuji Hospital, 028-8040 Kuji, Japan; Mikinogahara Dental Clinic, 034-0012 Towada, Japan; Division of Oral and Maxillofacial Surgery, Department of Oral and Maxillofacial Reconstructive Surgery, School of Dentistry, Iwate Medical University Dental and Oral Surgery, 020-8505 Morioka, Japan; Division of Oral and Maxillofacial Surgery, Department of Oral and Maxillofacial Reconstructive Surgery, School of Dentistry, Iwate Medical University Dental and Oral Surgery, 020-8505 Morioka, Japan; Division of Oral and Maxillofacial Surgery, Department of Oral and Maxillofacial Reconstructive Surgery, School of Dentistry, Iwate Medical University Dental and Oral Surgery, 020-8505 Morioka, Japan; Division of Oral Radiology, Department of Oral and Maxillofacial Reconstructive Surgery, School of Dentistry, Iwate Medical University, 020-8505 Morioka, Japan; Division of Oral and Maxillofacial Surgery, Department of Oral and Maxillofacial Reconstructive Surgery, School of Dentistry, Iwate Medical University Dental and Oral Surgery, 020-8505 Morioka, Japan

**Keywords:** dermoid cyst, oral floor, gourd-shaped

## Abstract

Dermoid cysts of the oral floor are rare cystic lesions of the oral cavity characterized by ectodermal-derived tissue. Normally, lesions appear round or ovoid on imaging. We report a rare case of a gourd-shaped dermoid cyst on the oral floor. An 11-year-old boy with a painless swelling in the oral floor and submental region was referred to our hospital, and magnetic resonance image presented a ground-shaped lesion through the bilateral mylohyoids. A cystic lesion was suspected, and excision was performed under general anesthesia. The excised lesion was then gourd. Pathological examination revealed a dermoid cyst. The patient’s postoperative course was uneventful, and no recurrence was observed.

## Introduction

Dermoid cysts are congenital cystic lesions arising from ectodermal epithelial tissue aberrantly entrapped during embryonic development, particularly due to malformations in the branchial arches or along embryonic fusion lines [1]. In some cases, similar cystic lesions may develop due to acquired factors, such as trauma or inflammation, which can implant and proliferate epithelial components within deeper tissues [2]. According to New *et al.*, ~1.6% of all dermoid cysts occur in the oral cavity, making this location uncommon [3]. These lesions are most commonly observed from infancy to adolescence with no significant sex predilection [3]. When dermoid cysts occur on the floor of the mouth, they must be clinically differentiated from ranulas [4]. Imaging typically reveals round or ovoid appearance [5]. We report a rare case of a gourd-shaped transmylohyoid dermoid cyst on the oral floor.

## Case report

An 11-year-old boy presented with a painless swelling on the oral floor that had gradually increased in size over the preceding year. In July 2023, he was referred to our department after consultation with a dental clinic. The patient’s medical and family histories were unremarkable. He was in good general health and well-nourished. Extraoral examination revealed swelling of the mental region. The mass was elastic, soft, fluctuating, and tender (Fig. 1A). Intraorally, a painless, elastic-soft swelling was observed at the midline of the oral floor. The overlying mucosa appeared normal (Fig. 1B). Panoramic radiography revealed no remarkable osseous changes (Fig. 1C). Axial magnetic resonance image (MRI) demonstrated a well-defined round lesion in the midline of the oral floor. On T1-weighted images, the lesion showed predominantly low signal intensity, with internal areas of intermediate signals. T2-weighted images revealed heterogeneous high-signal intensity. No abnormalities were observed in either submandibular gland (Fig. 2A and B). However, coronal and sagittal T2-weighted images showed a gourd-shaped and bilobed high-signal-intensity mass (Fig. 2C and D). A two-layered internal configuration was evident; the caudal portion demonstrated signal characteristics suggestive of serous components, while the cranial portion suggested ectodermal-derived material. The constricted portion of the lesion corresponded anatomically to the region between the bilateral sublingual glands and genioglossus muscles.

**Figure 1:**
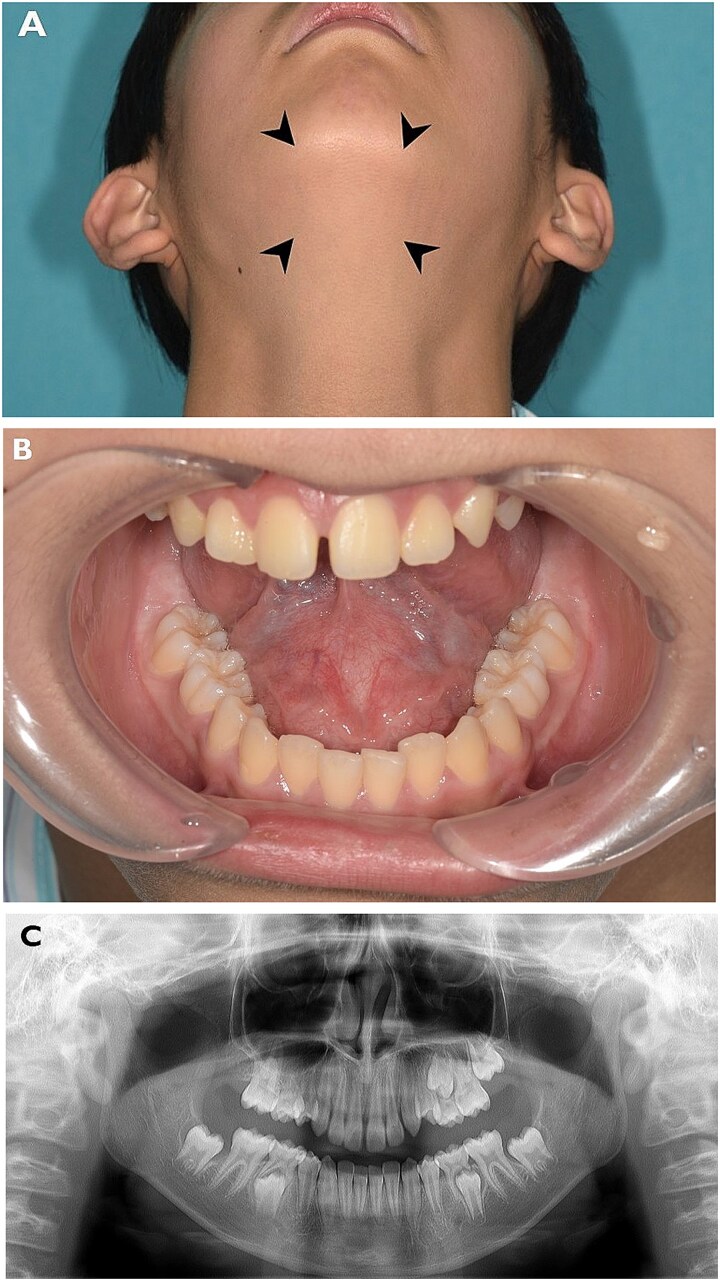
Clinical and radiographic findings at initial examination. (A) Extraoral photograph showing swelling in the mental region. (B) Intraoral photograph demonstrating a painless, elastic-soft swelling in the midline of the floor of the mouth, with normal overlying mucosa. (C) Panoramic radiograph showing no significant osseous changes.

**Figure 2:**
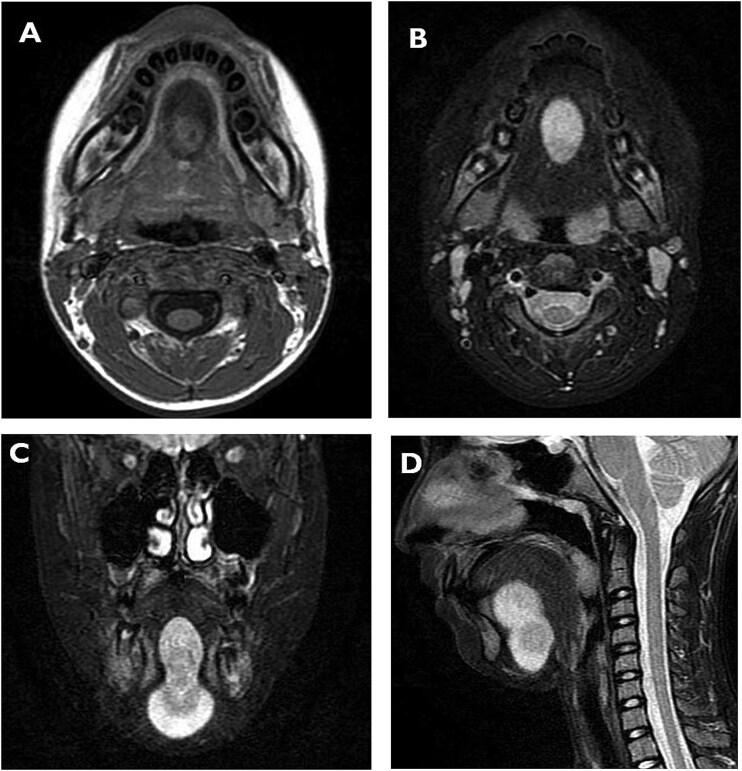
MRI of the lesion in the oral floor of the mouth. (A) Axial T1-weighted image showing a well-defined, round lesion with predominantly low signal intensity and areas of intermediate signal internally. (B) Axial T2-weighted image revealing heterogeneous high signal intensity; both submandibular glands appear normal. (C) Coronal T2-weighted image demonstrating a bilobed (‘gourd-shaped’) high-signal-intensity mass with a two-layered internal configuration: The caudal portion suggesting serous components and the cranial portion suggesting ectodermal-derived material. (D) Sagittal T2-weighted image showing the characteristic constriction between the bilateral sublingual glands and genioglossus muscles contributing to the bilobed morphology.

Suspecting a dermoid cyst, the lesion was completely excised from the oral floor under general anesthesia after providing sufficient explanation and obtaining consent from the patient. The excised specimen had a gourd-shaped structure (Fig. 3A). Histopathological examination revealed a relatively thin cyst wall lined with a stratified squamous epithelium. Beneath the epithelium, dense fibrous connective tissue was observed, which was covered externally by loose connective tissue. Skin appendages, including sebaceous glands and hair follicles, were identified within the cyst walls (Fig. 3B). The amount of keratinous material decreased toward the caudal portion of the cyst cavity, correlating with the high T2 signal intensity observed in this region. The final diagnosis was dermoid cyst. The patient’s postoperative course was uneventful, and no recurrence was observed.

**Figure 3:**
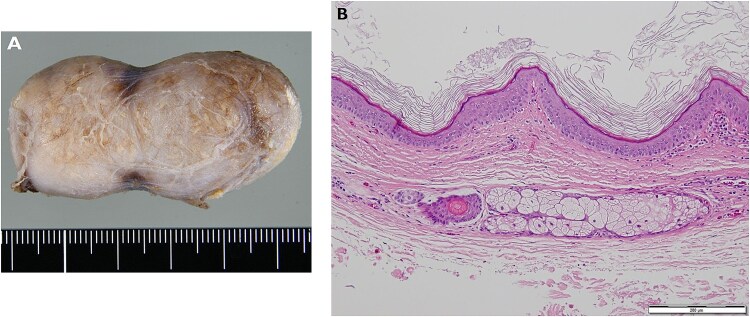
Macroscopic and histopathological findings of the excised lesion. (A) Gross specimen showing a gourd-shaped configuration consistent with preoperative imaging. (B) Histopathological section demonstrating a relatively thin cyst wall lined by stratified squamous epithelium. Beneath the epithelium, dense fibrous connective tissue is observed, covered externally by loose connective tissue. Skin appendages, including sebaceous glands and hair follicles, are present within the cyst wall.

## Discussion

Cystic lesions on the oral floor are the most common ranulas while dermoid cysts at this location are rare. According to New *et al.*, only 1.6% of all dermoid cysts occur in the oral cavity, making it an uncommon entity [3]. Dermoid cysts originate from ectodermal tissue trapped during embryonic development, often due to remnants of the branchial arches [6]. Acquired factors, such as trauma or inflammation, can also contribute to the implantation of epithelial tissue into deeper layers.

Dermoid cysts typically expand by compressing the surrounding tissues and present as slowly enlarging compressible masses. Complete surgical excision is the treatment of choice, and approaches include intraoral and extraoral methods [7]. The surgical method is usually determined according to lesion location, guided by classifications such as those proposed by Hagisaki [8]. The prognosis is generally excellent with complete excision, including that of the cyst capsule.

MRI findings of dermoid cysts usually show heterogeneous signal intensity on T1-weighted images and high signal intensity on T2-weighted images, often described as a ‘marble-like’ appearance [9]. In the present case, T1-weighted images demonstrated heterogeneous signal intensity, and T2-weighted images showed high-signal intensity; however, the characteristic ‘marble-like’ pattern was not observed. This is likely because the cyst cavity was filled with varying amounts of keratinous material. Both imaging and the excised specimen showed a gourd-shaped (bilobed) configuration, which may have resulted from compression and displacement of the genioglossus, geniohyoid muscles, and sublingual glands.

Gourd-shaped or ‘dumbbell-shaped’ dermoid cysts of the oral floor are extremely rare. A literature review revealed only two prior reports from Japan [10, 11]. To our knowledge, this is the third reported case. The rarity and distinctive morphology of this lesion highlight the importance of careful radiological assessment and surgical planning.
